# Feasibility of Oral Function Evaluation According to Dementia Severity in Older Adults with Alzheimer’s Disease

**DOI:** 10.3390/nu16070992

**Published:** 2024-03-28

**Authors:** Maki Shirobe, Ayako Edahiro, Keiko Motokawa, Shiho Morishita, Yoshiko Motohashi, Chiaki Matsubara, Masanori Iwasaki, Yutaka Watanabe, Hirohiko Hirano

**Affiliations:** 1 Research Team for Promoting Independence and Mental Health, Tokyo Metropolitan Institute for Geriatrics and Gerontology, Tokyo 173-0015, Japan; mshirobe@tmig.or.jp (M.S.); aedahiro514@gmail.com (A.E.); morishita947@gmail.com (S.M.); iwasaki@den.hokudai.ac.jp (M.I.); ywata@den.hokudai.ac.jp (Y.W.); h-hiro@gd5.so-net.ne.jp (H.H.); 2 School of Health Sciences, Meikai University, Chiba 279-8550, Japan; 3 Department of Dental Hygiene, University of Shizuoka, Shizuoka Junior College, Shizuoka 422-8021, Japan; 4 Department of Preventive Dentistry, Faculty of Dental Medicine, Graduate School of Dental Medicine, Hokkaido University, Hokkaido 060-8586, Japan; 5 Gerodontology, Department of Oral Health Science, Hokkaido University, Hokkaido 060-8586, Japan; 6 Dentistry and Oral Surgery, Tokyo Metropolitan Geriatric Hospital, Tokyo 173-0015, Japan

**Keywords:** oral function, functional assessment staging of Alzheimer’s disease, oral diadochokinesis, swallowing function, dementia, oral health, long-term care

## Abstract

Oral function evaluation in older adults with dementia is important for determining appropriate and practical dietary support plans; however, it can be challenging due to their difficulties in comprehending instructions and cooperating during assessments. The feasibility of oral function evaluation has not been well studied. This cross-sectional study aimed to determine the feasibility of oral function evaluation in older adults with Alzheimer’s disease (AD) according to Functional Assessment Staging of Alzheimer’s Disease (FAST) stages. In total, 428 older adults with AD (45 men and 383 women; mean age: 87.2 ± 6.2 years) were included. Multilevel logistic regression models were used to examine the prevalence of participants who were unable to perform oral function evaluations, including oral diadochokinesis (ODK), repeated saliva swallow test (RSST), and modified water swallow test (MWST). In comparison to the reference category (combined FAST stage 1–3), FAST stage 7 was associated with the infeasibility of ODK (adjusted odds ratio, 95% confidence interval = 26.7, 4.2–168.6), RSST (5.9, 2.2–16.1), and MWST (8.7, 1.6–48.5, respectively). Oral function evaluation is difficult in older adults with severe AD. Simpler and more practical swallowing function assessments and indicators that can be routinely observed are required.

## 1. Introduction

Almost 30 years ago, individuals with dementia had a survival period of 4 years following disease onset [1]. However, today, those with dementia experience a longer disease duration and survival period despite a decline in activities of daily living (ADLs) [2,3]. For instance, the total duration of Alzheimer’s disease (AD) in a 70-year-old patient with AD-related dementia is 20 years, with approximately 3 years in moderate-to-severe dementia. Similarly, a female patient who develops dementia at 65 years of age may experience moderate-to-severe dementia for approximately 6 years [4]. Therefore, the duration of care for patients with moderate-to-severe dementia is longer today than in the past, making care planning essential, especially for patients with dementia in long-term care. A particular issue in patients with dementia in long-term care is that malnutrition is often observed. The results of meta-analyses including studies of regions in Europe or South Asia pooled the prevalence of malnutrition in this population at 57% [5]. This suggests that nutritional management is particularly important in planning care for patients with dementia. Evaluation of oral function, including eating and swallowing abilities, in patients with dementia is important for determining appropriate and practical dietary support plans. Oral function has been shown to be related to nutritional intake [5,6], food forms [7,8], and nutritional status [9,10]. A study conducted in nursing home residents worldwide aged ≥65 years from Europe and North America reported an association between the presence of dysphagia, evaluated using responses of nursing homes staff and nutritional intake [6]. In contrast, a Japanese piece of research with older adults in long-term care at home or nursing homes showed an association between dysphagia, examined using cervical auscultation of swallowing sounds of 3 mL of water with a stethoscope and malnutrition [7]. Regarding the number of teeth, a cross-sectional study showed a lower body mass index (BMI) in nursing home residents with dementia without molar occlusion than in those with molar occlusion [10].

It should be noted that oral function evaluation in older patients with dementia presents challenges due to difficulties they encounter in comprehending instructions and cooperating while undergoing these assessments [11,12,13,14]. Previous studies have assessed swallowing function in hospitalized older patients with dementia using video fluorographic examination of swallowing (VF) [15,16]. In addition, oral function of institutionalized older patients with dementia has been assessed using simple measures, such as interviews with caregivers, questionnaires, and observation of the oral cavity [9,17,18]. In other studies, oral function evaluation included multiple swallowing assessments for mild cognitive impairment (MCI) and mild dementia [19] or caregivers’ assessments of feeding and swallowing functions were performed using the Clinical Dementia Rating [20]. However, no reports have objectively evaluated eating and swallowing functions in patients with severe dementia. Further, dementia severity was broadly defined in previous studies [10] and should be examined in more detail.

Therefore, we conducted a cross-sectional study to assess the feasibility of oral function evaluation in older patients with AD based on the stages outlined by the Functional Assessment Staging of Alzheimer’s Disease (FAST) [21].

## 2. Materials and Methods

### 2.1. Study Setting, Design, and Participants

This study is a secondary analysis of data obtained from the Akita–Omorimachi study [22,23]. The Akita–Omorimachi study is an epidemiological study conducted to determine the factors related to health longevity of older adults in long-term care in the Omorimachi area of Yokote City, Akita Prefecture, Japan. Data pertaining to the survey items such as medical history, numbers of medication, ADL, height, weight, muscle mass, cognitive function, nutritional status, and oral function were collected annually.

The eligibility criteria for the current study were delineated as follows: The inclusion criteria comprised the following: (i) those who participated in the Akita–Omorimachi study spanning from 2015 to 2020 and (ii) those with a diagnosis of AD extracted from their medical records, subsequently verified by a psychiatrist. The exclusion criteria were as follows: (i) those who were unable to intake food orally and (ii) those with incomplete data.

The type of dementia was specified as a survey item within the Akita–Omorimachi Study from 2015. The survey was transcribed from the participant’s medical record by staff at each facility. Subsequently, these records for each participant across the survey year (between 2015 and 2020) were in aggregate and utilized as cross-sectional data for this study. Given the nature of this investigation as a derivative study from the Akita–Omorimachi Study, no specific sample size calculation was performed. All individuals meeting the eligibility criteria from the original Akita–Omorimachi Study cohort were included in the current analysis.

The methodology employed in this study received approval from the ethics committee of the Tokyo Metropolitan Institute for Geriatrics and Gerontology (approval numbers: 26, R17-15, and 37; approval dates: 17 June 2013, 8 September 2017, and 13 November 2019). All activities adhered to the guidelines outlined in the Declaration of Helsinki concerning experiments involving human participants. Explicit written informed consent was acquired from either the study participants or their respective families.

### 2.2. Assessment of Dementia Severity

The severity of dementia was assessed by two certified geriatric nutritional physicians accredited by the Japan Geriatrics Society using FAST. FAST is a validated method used to assess the extent of impairment caused by dementia, dividing it into 16 distinct levels [21]. These 16 FAST levels are further grouped into 7 major stages, each representing different degrees of impairment: “1, normal aging; 2, possible MCI; 3, MCI; 4, mild dementia; 5, moderate dementia; 6, moderately severe dementia; and 7, severe dementia”. Because only 1 (0.2%), 18 (3.8%), and 12 (2.5%) participants were in FAST stages 1, 2, and 3, respectively, they were included in a single combined category (i.e., combined FAST stage 1–3).

### 2.3. Assessment of Oral Function Evaluation

The feasibility of oral function evaluation was determined by whether a score could be recorded when the measurement was performed as follows: Oral function was evaluated by either dentists or dental hygienists, with all investigators undergoing thorough training beforehand to ensure consistent examination standards.

Objective evaluations were performed using oral diadochokinesis (ODK), repeated salivary swallow test (RSST), and modified water swallow test (MWST).

In the ODK test, participants repeated the monosyllable /ta/ for 5 s rapidly, and the number of times it was pronounced was recorded [24]. A digital counter (T.K.K. 3350 digital counter; Takei Rika Kikai Kikai, Niigata, Japan) was utilized to perform the ODK test, which is an index used to assess the motor function of the lips and tongue. In this study, we focused on the monosyllable /ta/, which is related to the motor function of the anterior part of the tongue.

In the RSST, the participants are asked to swallow their saliva repeatedly for a duration of 30 s [25]. This test measures the number of swallows performed by a participant within a definite period. The assessment is conducted by gently palpating the laryngeal ridge, a prominent structure in the neck, to detect each swallow. It is a simple method used to assess swallowing function.

The MWST involves injecting 3 mL of cold water into the bottom of the mouth with a 5 mL syringe and instructing the participants to swallow [26]. To better assess the swallowing status, a stethoscope is placed on the throat to auscultate the swallowing and breathing sounds in the pharynx [27]. This is quantified using a five-point scale based on the participant’s ability to swallow, occurrence of swallowing, and associated breathing difficulties or hoarseness.

### 2.4. Data Collection for Basic Information

Data pertaining to demographic factors (sex and age), comorbidities (Parkinson’s disease, neurological disease, respiratory disease, stroke, cardiovascular disease, diabetes mellitus, and cancer), body weight, body height, and nutritional status were collected using questionnaires. BMI was calculated as weight in kilograms divided by height in meters squared.

Nutritional status was assessed using the Mini Nutritional Assessment^®^-Short Form (MNA^®^-SF) [28], which consists of six questions as follows: (i) food intake decline over the last 3 months (scoring: 0 = severe decrease; 1 = moderate decrease; and 2 = no decrease in food intake); (ii) weight loss during the last 3 months (0 = weight loss > 3 kg; 1 = does not know; 2 = weight loss between 1 and 3 kg; and 3 = no weight loss); (iii) mobility (0 = bed or chair bound; 1 = able to get out of bed/chair but does not go out; and 2 = able to go out); (iv) psychological stress or acute disease in the last 3 months (0 = yes; 2 = no); (v) neuropsychological problems (0 = severe dementia or depression; 1 = mild dementia; and 2 = no psychological problems); and (vi) BMI (0 = BMI < 19 kg/m^2^; 1 = 19 ≤ BMI < 21 kg/m^2^; 2 = 21 ≤ BMI < 23 kg/m^2^; and 3 = BMI ≥ 23 kg/m^2^). The total scores for the six questions pertain to the nutritional status, with lower scores indicating malnutrition (range: 0–14). A score of 0–7 indicated malnutrition, 8–11 a risk of malnutrition, and 12–14 good nutritional status.

### 2.5. Statistical Analysis

Initially, the characteristics of participant records were delineated based on the FAST stages. The Shapiro–Wilk test was used to assess the normal distribution of continuous variables.

Subsequently, the association between FAST stages and feasibility of oral function evaluation was evaluated using a multilevel logistic regression analysis. Given that the record served as the unit of analyses, a multilevel model was applied to mitigate correlations among records within the same participant. The macro-level variable included the survey year, while micro-level variables included the remaining variables. Exposure variables included FAST stages (combined FAST stages 1–3 were set as the reference category). The outcome variable was the feasibility of oral function evaluation (coding; 0: possible, 1: impossible to perform the evaluation). Both univariable and multivariable analyses were performed to estimate odds ratios (ORs) along with 95% confidence intervals (CIs) for the infeasibility of oral function evaluation. Covariates for multivariable analysis were selected based on a priori knowledge [23] as follows: age, sex, BMI, and number of comorbidities.

The software for statistical analyses was the IBM SPSS Statistics for Windows software (version 29.0; IBM Corp., Armonk, NY, USA). A *p* value < 0.05 was defined as statistically significant.

## 3. Results

Between 2015 and 2020, we acquired 2370 records corresponding to 962 individuals. Among these, 521 records pertained to individuals with AD. We eliminated 60 records from individuals who were unable to orally ingest and 33 records from those with incomplete datasets. Ultimately, our analysis included 428 records from 211 individuals. These comprised 31, 19, 59, 143, and 176 records from participants categorized with FAST stages 1–3, 4, 5, 6, and 7, respectively. The summary characteristics of participants by the FAST stage are presented in Table 1. The participants were mostly female (89.5%), with a median age of 88 years. Overall, the infeasibility percentages were 27.3%, 34.1%, and 11.0% for ODK, RSST, and MWST, respectively. The body composition of the participants was as follows: median height of 145.0 cm, median weight of 45.5 kg, and median BMI of 21.6 kg/m^2^. In addition to dementia, the most prevalent comorbidities were cardiovascular disease (53.0%), stroke (18.9%), and diabetes mellitus (15.2%). Furthermore, nutritional status was evaluated using MNA-SF, revealing that 18.7% participants were classified as normal, 47.9% were at risk, and 33.4% were undernourished. Moreover, the prevalence of malnutrition, as determined by MNA-SF in correlation with FAST, was 9.7% in participants with FAST stages 1–3, 15.8% in those with FAST stage 4, 16.9% in those with FAST stage 5, 26.6% in those with FAST stage 6, and 50.6% in those with FAST stage 7.

Table 2 shows the multilevel logistic regression results. AD severity, as gauged by the FAST stage, exhibited associations with the feasibility of ODK, RSST, and MWST evaluations. The univariate ORs (95% CI) for inability to assess ODK in participants with FAST stages 4–7 (compared with those with a combined FAST stages 1–3) were 0.00 (0.00–0.00), 0.49 (0.04–6.78), 3.28 (0.52–20.66), and 32.46 (5.42–194.35), respectively. The corresponding figures for infeasibility in RSST were 0.21 (0.02–2.18), 0.17 (0.03–0.92), 1.05 (0.39–2.84), and 7.61 (2.92–19.80), respectively. Additionally, the corresponding figures for infeasibility in MWST were 0.00 (0.00–0.00), 0.00 (0.00–0.00), 0.00 (0.00–0.00), and 13.06 (1.95–87.42), respectively. In addition, the multivariate model included sex, age, BMI and number of comorbidities as covariates. The multivariable adjusted ORs (95% CIs) for infeasibility in ODK across FAST stages 4 to 7 (compared with the combined FAST stage 1–3) were 0.00 (0.00–0.00), 0.38 (0.02–6.78), 2.94 (0.44–19.70), and 26.67 (4.22–168.61), respectively. The corresponding values for infeasibility in MWST were 0.00 (0.00–0.00), 0.00 (0.00–0.00), 0.22 (0.02–2.42), and 8.70 (1.56–48.53), respectively. Furthermore, there was a nonlinear relationship between the FAST stage and the feasibility of RSST. Relative to the combined FAST stage 1–3, ORs (95% CIs) for infeasibility in RSST were 0.15 (0.03–0.82) for FAST stage 5 and 5.94 (2.20–16.05) for FAST stage 7.

## 4. Discussion

The results of this study showed a trend toward failure of performance of the ODK test, RSST, and MWST in participants with severe dementia. In addition, RSST showed a nonlinear association with the feasibility of assessments according to dementia severity. To the best of our knowledge, this is the first study to determine the feasibility of oral function evaluations in older adults with AD according to the severity of dementia as assessed by FAST.

ODK was not feasible in a higher percentage of participants in FAST stage 7. The results were similar to those of a previous study of older adults requiring long-term care, although the method of assessing the severity of dementia was different [29]. In AD, speech apraxia occurs as the disease progresses [30]. Therefore, it is conceivable that more participants with FAST stage 7, in which pronouncing a predetermined syllable becomes more difficult, were unable to perform ODK. In addition, most individuals with severe dementia were unable to comprehend examiners’ instructions because of cognitive decline. The difficulty in pronouncing the same syllable consecutively, even when assessing only the clarity of pronunciation, underlies this result. ODK is a measure of lip and tongue motor functions but is also associated with dysphagia [31] and tongue coating [32]. Therefore, if it is difficult to assess persons with severe dementia, other assessment methods should be considered depending on the purpose of assessment.

RSST is considered to be a simple assessment tool that can be performed by caregivers and family members who are not experts [33]. However, significantly more individuals with severe dementia were unable to undergo RSST. The evaluation method of RSST requires individuals to voluntarily repeat saliva swallowing without the use of water, following verbal instructions. Therefore, many individuals with FAST stage 7 could not perform this test because of cognitive function and ADL decline. Similar results were obtained with the MWST, which is used in screening for dysphagia [26]; however, it is not frequently used in nursing home facilities as participants often drink water with a thickening agent. Therefore, a simpler evaluation method for swallowing function is required for individuals with severe dementia. In cases where fluid intake is difficult, tests that use semisolid foods may be performed [34,35]. In addition, as a simple indicator, it is necessary to assess the rinsing ability, which has been shown to be associated with dysphagia [36,37], and monitor daily eating habits [38].

In contrast, RSST was not feasible in a lower percentage of participants with FAST stage 5 than of participants with combined FAST stage 1–3. One reason for this was that some participants with the combined FAST stage 1–3 were resistant to placing hands on the pharyngeal region. Therefore, more participants with combined FAST stage 1–3 were unable to perform the test than those with FAST stage 5. Although the manifestation rate of agitation symptoms is not proportional to the severity of dementia and refusal of care is rare in participants with FAST stage 1–3 [39], performing RSST is different from usual care. It is also possible that the surveyor, rather than the caregiver who routinely provides care, inspected the individuals, which may have contributed to their refusal. Thus, physical contact and a lack of trust in the investigator may be related. This is different from the reason of inability of participants with severe dementia to undergo the examination because of poor comprehension.

In the early stages of AD, dysphagia is more likely to occur because of oral dysfunction, such as decreased tongue motor and masticatory functions [36] and challenges in the oral phase of swallowing (i.e., the first stage of the swallowing process [40]), such as delayed swallowing reflexes. Although oral phase challenges are associated with longer mealtimes and the risk of malnutrition [41,42], they do not pose clinical challenges and are often unnoticed by caregivers [15]. In moderate and severe AD, dysphagia is caused by cognitive and motor decline [43]. Aspiration pneumonia is a common cause of death, especially in older adults with severe dementia, and the management of dysphagia is important to prevent aspiration pneumonia [44]. Furthermore, we suspect that the inability to properly assess oral function may have influenced the selection of appropriate food forms. Food form is associated with energy intake [45] and selecting the appropriate food form contributes to improved nutritional status [46]. In nursing homes, assessing swallowing function is difficult because of the absence of specialist staff. In clinical settings, nursing staff should be able to easily screen and change food forms [47]. Therefore, in both severities of dementia, the evaluation of oral function, including swallowing function, is important. The results of this study showed that several swallowing tests could not be performed in adults with dementia, especially those with severe dementia in FAST stage 7. Changes in routine care, such as observation during meals, should be performed, in addition to oral function evaluation, because assessment using questionnaires may be impossible in this condition.

This study has several limitations. First, because of the small number of participants with MCI based on age appropriateness, FAST stages 1, 2, and 3 were combined into one category for analysis. Furthermore, the number of participants was insufficient to estimate the OR for the feasibility of performing ODK and MWST, even in participants with FAST stages 4 and 5. Although this study sheds light on challenges related to oral function evaluation in older adults with severe dementia, analogous issues in older adults with milder dementia warrant further investigation in future studies. Second, this study did not evaluate the reasons for the inability to examine oral function in detail. Reasons for non-acceptance of examination included physical refusal, decline in ADL, and inability to understand the examination procedure. In particular, a participant’s cognitive function, which should be considered while selecting an assessment, is very different when the reason for not allowing the assessment is physical refusal or inability to understand the instructions. Therefore, further studies should focus on the reasons for the impossibility of the assessment of oral function. Third, oral function evaluations performed in this study were limited in terms of evaluation methods. In previous studies on dysphagia in AD [48], dietary observations [49] and VF [50] were used to evaluate eating and swallowing functions. Many other evaluation methods [51,52] require the consumption of solid foods or sufficient time for investigation. Although they are useful for screening, such as in the selection of food forms, they are difficult to use in epidemiological studies, such as this one. As the study targeted individuals requiring care at multiple facilities, a method that could be employed in a limited timeframe was required, which would be relatively easy to perform by the study participants.

This study was conducted in several nursing homes and designed to examine the most common form of AD in adults with dementia. As this study was not performed in a specific facility and the data were obtained from an observational cohort study, the impact of individual facilities is expected to be small, with limited variation among facilities and regions. Therefore, we contend that the findings from this study will be beneficial for performing oral function evaluation in other facilities for older adults requiring nursing care.

## 5. Conclusions

The results of this study indicate that oral function evaluation is difficult in older adults with severe AD. The reasons for the difficulty in performing RSST and MWST were varied, depending on the severity of dementia. Appropriate implementation of the assessment requires an understanding of the instructions and the voluntary cooperation of older adults with dementia. Therefore, it is necessary to consider simpler and more practical assessments of swallowing function and indicators that can be routinely assessed.

## Figures and Tables

**Table 1:** Case characteristics according to FAST.

	Overall (*n* = 428)	Combined FAST Stage 1–3 (*n* = 31)	FAST Stage 4 (*n* = 19)	FAST Stage 5 (*n* = 59)	FAST Stage 6 (*n* = 143)	FAST Stage 7 (*n* = 176)
Feasibility of oral function evaluation						
ODK, impossible	117 (27.3)	1 (3.2)	0 (0)	2 (3.4)	16 (11.2)	98 (55.7)
RSST, impossible	146 (34.1)	6 (19.4)	1 (5.3)	3 (5.1)	31 (21.7)	105 (59.7)
MWST, impossible	47 (11.0)	1 (3.2)	0 (0)	0 (0)	2 (1.4)	44 (25.0)
Other characteristics						
Sex, female	383 (89.5)	30 (96.8)	17 (89.5)	50 (84.7)	125 (87.4)	161 (89.5)
Age, years	88 (83–91)	90 (86–91)	86 (82–89)	87 (81–92)	86 (83–90)	88.5 (84–92)
Height, cm	145.0 (140.0–150.0)	143.0 (140.2–150.0)	145.4 (143.0–155.6)	145.0 (140.5–151.0)	145.0 (140.0–150.0)	144.0 (138.0–150.0)
Weight, kg	45.5 (39.4–52.2)	45.5 (36.5–55.8)	46.8 (40.2–52.8)	48.8 (43.5–55.2)	47.7 (42.0–53.6)	42.5 (37.3–47.8)
BMI, kg/m^2^	21.6 (18.8–24.4)	22.4 (18.1–26.7)	21.6 (18.6–24.3)	22.6 (20.5–25.8)	22.6 (19.8–25.0)	20.5 (18.4–22.8)
Medical history						
Cancer	27 (6.3)	0 (0)	1 (5.3)	6 (10.2)	11 (7.7)	9 (5.1)
Cardiovascular disease	227 (53.0)	21 (67.7)	11 (57.9)	32 (54.2)	69 (48.3)	94 (53.4)
Stroke	81 (18.9)	8 (25.8)	1 (5.3)	6 (10.2)	27 (18.9)	39 (22.2)
Diabetes mellitus	65 (15.2)	4 (12.9)	6 (31.6)	5 (8.5)	14 (9.8)	36 (20.5)
Neurological disorders	6 (1.4)	2 (6.5)	0 (0)	0 (0)	4 (2.8)	0 (0)
Parkinson’s disease	10 (2.3)	1 (3.2)	1 (5.3)	2 (3.4)	2 (1.4)	4 (2.3)
Respiratory diseases	28 (6.5)	0 (0)	2 (10.5)	5 (8.5)	8 (5.6)	13 (7.4)
MNA^®^-SF						
Normal	80 (18.7)	12 (38.7)	8 (42.1)	19 (32.2)	36 (25.2)	5 (2.8)
At risk	205 (47.9)	16 (51.3)	8 (42.1)	30 (50.8)	69 (48.3)	82 (46.6)
Malnutrition	143 (33.4)	3 (9.7)	3 (15.8)	10 (16.9)	38 (26.6)	89 (50.6)

Data are presented as *n* (%) or the median (IQR). FAST, Functional Assessment Staging of Alzheimer’s Disease; ODK, oral diadochokinesis; RSST, Repeated Salivary Swallow Test; MWST, Modified Water Swallowing Test; BMI, body mass index; MNA^®^-SF, Mini Nutritional Assessment^®^-Short Form; IQR, interquartile range.

**Table 2:** ORs for feasibility of oral function evaluation according to FAST.

	Outcome Variables								
Exposure Variable	ODK (0: Possible, 1: Impossible)			RSST (0: Possible, 1: Impossible)			MWST (0: Possible, 1: Impossible)		
Univariable model	OR	95% CI	*p*-value	OR	95% CI	*p*-value	OR	95% CI	*p*-value
FAST 1–3	Ref.			Ref.			Ref.		
FAST 4	0.00	0.00–0.00	1.000	0.21	0.02–2.18	0.193	0.00	0.00–0.00	0.998
FAST 5	0.49	0.04–6.78	0.590	0.17	0.03–0.92	0.040	0.00	0.00–0.00	0.994
FAST 6	3.28	0.52–20.66	0.205	1.05	0.39–2.84	0.925	0.00	0.00–0.00	0.976
FAST 7	32.46	5.42–194.35	<0.001	7.61	2.92–19.80	<0.001	13.06	1.95–87.42	0.008
Multivariable model *	Adjusted OR	95% CI	*p*-value	Adjusted OR	95% CI	*p*-value	Adjusted OR	95% CI	*p*-value
FAST 1–3	Ref.			Ref.			Ref.		
FAST 4	0.00	0.00–0.00	1.000	0.18	0.02–1.65	0.129	0.00	0.00–0.00	0.998
FAST 5	0.38	0.02–6.78	0.511	0.15	0.03–0.82	0.029	0.00	0.00–0.00	0.996
FAST 6	2.94	0.44–19.70	0.266	0.89	0.32–2.52	0.830	0.22	0.02–2.42	0.216
FAST 7	26.67	4.22–168.61	<0.001	5.94	2.20–16.05	<0.001	8.70	1.56–48.53	0.014

* Except for sex, age, body mass index, and number of comorbidities, ORs and CIs of being positive are presented. ODK, oral diadochokinesis; RSST, Repeated Salivary Swallow Test; MWST, Modified Water Swallowing Test; CI, confidence interval; FAST, Functional Assessment Staging of Alzheimer’s Disease; OR, odds ratio; Ref., reference.

## Data Availability

The data presented in this study are accessible upon request from the corresponding author. However, the data are not publicly available. The reason is that there are ethical and legal restrictions enforced by the Ethics Committee of the Tokyo Metropolitan Institute for Geriatrics and Gerontology.
